# Tetrahydrobiopterin Plays a Functionally Significant Role in Lipogenesis in the Oleaginous Fungus *Mortierella alpina*

**DOI:** 10.3389/fmicb.2020.00250

**Published:** 2020-02-20

**Authors:** Hongchao Wang, Chen Zhang, Haiqin Chen, Zhennan Gu, Jianxin Zhao, Hao Zhang, Yong Q. Chen, Wei Chen

**Affiliations:** ^1^State Key Laboratory of Food Science and Technology, Jiangnan University, Wuxi, China; ^2^School of Food Science and Technology, Jiangnan University, Wuxi, China; ^3^National Engineering Research Center for Functional Food, Jiangnan University, Wuxi, China

**Keywords:** *Mortierella alpine*, tetrahydrobiopterin, GTP cyclohydrolase I, NADPH, lipogenesis

## Abstract

Tetrahydrobiopterin (BH_4_) is well-known as a cofactor of phenylalanine hydroxylase (PAH) and nitric oxide synthase (NOS), but its exact role in lipogenesis is unclear. In this study, the GTP cyclohydrolase I (GTPCH) gene was overexpressed to investigate the role of BH_4_ in lipogenesis in oleaginous fungus *Mortierella alpina*. Transcriptome data analysis reveal that GTPCH expression was upregulated when nitrogen was exhausted, resulting in lipid accumulation. Significant changes were also found in the fatty acid profile of *M. alpina* grown on medium that contained a GTPCH inhibitor relative to that of *M. alpina* grown on medium that lacked the inhibitor. GTPCH overexpression in *M. alpina* (the *MA-GTPCH* strain) led to a sevenfold increase in BH_4_ levels and enhanced cell fatty acid synthesis and poly-unsaturation. Increased levels of nicotinamide adenine dinucleotide phosphate (NADPH) and upregulated expression of NADPH-producing genes in response to enhanced BH_4_ levels were also observed, which indicate a novel aspect of the NADPH regulatory mechanism. Increased BH_4_ levels also enhanced phenylalanine hydroxylation and nitric oxide synthesis, and the addition of an NOS or a PAH inhibitor in the *MA-GTPCH* and control strain cultures decreased fatty acid accumulation, NADPH production, and the transcript levels of NADPH-producing genes. Our research suggests an important role of BH_4_ in lipogenesis and that the phenylalanine catabolism and arginine–nitric oxide pathways play an integrating role in translating the effects of BH_4_ on lipogenesis by regulating the cellular NADPH pool. Thus, our findings provide novel insights into the mechanisms of efficient lipid biosynthesis regulation in oleaginous microorganisms and lay a foundation for the genetic engineering of these organisms to optimize their dietary fat yield.

## Introduction

Tetrahydrobiopterin (BH_4_) is well-known as an essential cofactor of animal nitric oxide synthase (NOS) and monooxygenases that require pteridine, including tyrosine hydroxylase, tryptophan hydroxylase, and phenylalanine hydroxylase (PAH) (Kaufman, 1993). PAH is responsible for the irreversible hydroxylation of phenylalanine to tyrosine as determined by BH_4_, which is the rate-limiting step in phenylalanine catabolism. BH_4_ is also essential for controlling the electron flow in NOS, which donates an electron and a proton to versatile intermediates in the reaction cycle of arginine/oxygen to citrulline/nitric oxide (NO) (Kappock and Caradonna, 1996; Neckameyer et al., 2007). BH_4_ is a major determinant of whether NOS produces NO or superoxide and thus also plays a key role in regulating cellular oxidative stress (Schulz et al., 2008). Studies have suggested that BH_4_ plays an important role in lipogenesis in higher organisms, including synthesis of long-chain polyunsaturated fatty acids (PUFAs), unsaturation or omega oxidation of long-chain PUFAs, and incorporation of fatty acids into phospholipids (Kaufman, 1967, 1993; Forrest and Van Baalen, 1970; Giovannini et al., 1995; Rudzite et al., 1998; Moseley et al., 2002). The role of BH_4_ may be significant in lipogenesis, but no direct biochemical evidence has confirmed the importance of BH_4_ in lipogenesis.

*Mortierella alpina* is a well-known lipid-producing fungus that produces a high level of PUFAs (Ji et al., 2014; Wang H. et al., 2016). PUFAs are the structural components of membrane phospholipids and the major precursors of prostaglandins, thromboxanes, and leukotrienes that play vital roles in cell signaling (Ji and Huang, 2018). Understanding the mechanisms by which high-efficiency lipid synthesis can be achieved in this oleaginous fungus could be instrumental in the application of single-cell oils as dietary supplements. Nicotinamide adenine dinucleotide phosphate (NADPH) is the limiting factor and a critical reducing agent in lipid biosynthesis (Wang et al., 2013). Its important sources are malic enzyme (ME) and the pentose phosphate pathway (PPP) (Dourou et al., 2018); however, evidence has shown that some NADPH may also be generated by isocitrate dehydrogenase (IDH) in the TCA cycle and folate metabolism (Fan et al., 2014; Chen et al., 2015). Although some of the genes essential for lipogenesis in *M. alpina* have been studied at the molecular level, the molecular mechanism of fatty acid synthesis and unsaturation in *M. alpina* in particular and in oleaginous microbes in general is still not well-understood (Michaelson et al., 1998; Sakuradani et al., 1999a, b,c, 2005).

Because most fungal genomes lack orthologous genes involved in BH_4_ biosynthesis (Wang H. et al., 2011; Wang et al., 2013), BH_4_ biosynthesis and function have not been explored in the kingdom Fungi. In our previous study, we sequenced the whole genome of *M. alpina* to investigate the presence of putative genes for BH_4_ synthesis (Wang L. et al., 2011). Our laboratory is the first to comprehensively characterize the BH_4_ *de novo* biosynthesis, salvage, and regeneration pathways in a fungus (Wang H. et al., 2011; Wang et al., 2013; Wang H. C. et al., 2016). However, the answers to the rather basic issues, such as the reason *M. alpina* needs to synthesize BH_4_ and the exact role of BH_4_ in fungi, have remained elusive. Our previous work characterized the BH_4_-dependent PAH in *M. alpina* and suggested that BH_4_ is important in lipogenesis in this fungus (Wang et al., 2013). The genome sequence of this fungus also suggests the presence of a BH_4_-dependent NOS. Thus, *M. alpina* could be a model organism to study the function of BH_4_ in lipogenesis.

GTP cyclohydrolase I (GTPCH) is responsible for the conversion of GTP to dihydroneopterin triphosphate, which is the rate-determining step for BH_4_ biosynthesis (Wang et al., 2013). GTPCH overexpression is a suitable approach to enhance BH_4_ biosynthesis in transgenic mice, and it can reduce endothelial dysfunction and atherosclerosis (Alp et al., 2004), accelerate refractory wound healing in diabetes (Tie et al., 2009), repair kidney injury (Wang et al., 2008), restore ischemic preconditioning during hyperglycemia (Ge et al., 2011), and attenuate blood pressure progression by regulating NOS activity (Du et al., 2008). However, it is unclear whether GTPCH overexpression affects the fatty acid content in oleaginous microorganisms, and to date, GTPCH has never been overexpressed in a microorganism.

In this study, GTPCH overexpression was induced in *M. alpina* to enhance the availability of BH_4_ and enable the assessment of the roles of BH_4_ in lipogenesis. NADPH generation, phenylalanine hydroxylation, and NO synthesis were also investigated to explore the regulatory mechanism of intracellular BH_4_ biosynthesis on lipogenesis. This study provides novel insights into the mechanisms of efficient lipid biosynthesis in oleaginous microorganisms and lays a foundation for the genetic engineering of these organisms to optimize the production of dietary fat.

## Materials and Methods

### Strains and Media

*Mortierella alpina* (ATCC 32222) was cultured in Kendrick medium for 8 days as described earlier (Wang et al., 2013). The mycelia were collected by filtration through a sterile cheesecloth after 8 days of cultivation and frozen immediately in liquid nitrogen for the extraction of DNA, RNA, NADPH, lipid, and other metabolites. The ammonium concentration of the culture filtrate was measured using the indophenol test (Chaney and Marbach, 1962).

### Transcriptome Data Analysis

RNA-sequencing data obtained using the Illumina GA IIx sequencing platform have been deposited in the Sequence Read Archive database^1^ (accession numbers SRR1638088, SRR1638089, SRR1638091, SRR1638092, SRR1638093, and SRR1638095) (Chen et al., 2015). The reads were trimmed from the end to 75 bp, mapped to the coding sequences extracted from the annotated *M. alpina* genome, and analyzed as previously described (Chen et al., 2015). Transcript abundances were calculated using Cufflinks version 0.9.3 with the corresponding genomic regions of annotated genes used as input reference annotation. The output normalized expression values in fragments per kilobase of exon per million fragments mapped (FPKM) were used for further comparative analysis (Mortazavi et al., 2008; Trapnell et al., 2010).

### Effects of a GTPCH Inhibitor on Lipid Synthesis

The GTPCH inhibitor 2, 4-diamino-6-hydroxypyrimidine (DAHP) inhibits GTPCH by direct competition with substrate GTP, which has been extensively used to investigate the function of BH_4_ (Kolinsky and Gross, 2004). In this study, the fungal cultures were grown in 50 mL of Kendrick medium containing 5 mM DAHP at 28°C for 8 days. The mycelia collected on day 8 were used to extract lipids. The Bligh and Dyer method was used for lipid extraction under acidic conditions with heneicosanoic acid and pentadecanoic acid added as internal standards (Wang L. et al., 2011). Fatty acid methyl esters were analyzed by gas chromatography (GC-2010; Shimadzu, Japan) using a DB-WAXetr column (30 m × 0.32 mm; film thickness, 0.25 μm). The temperature program was as follows: 120°C for 3 min, ramp to 190°C at 5°C/min, ramp to 220°C at 4°C/min, and hold for 20 min. Nitrogen was used as the carrier gas at a constant flow rate of 3 mL/min. This experiment was replicated three times.

### GTPCH Cloning and Overexpression in *M. alpina*

TRIzol reagent (Invitrogen) was used to extract total RNA. RNA was reverse transcribed to cDNA using the iScript Select cDNA synthesis kit (Bio-Rad) according to the manufacturer’s instructions. The GTPCH gene (JF746874) was amplified using the primers (GTPCH-F/R) presented in Supplementary Table 1. The PCR conditions were as follows: denaturing at 94°C for 0.5 min, annealing at 50°C for 0.5 min, and amplification at 68°C for 1 min (25 cycles). The PCR products were purified and cloned into the binary vector pBIG2-ura5s-IT1 (Chen et al., 2015), and the resulting GTPCH overexpression plasmid was named pBIG2-ura5s-GTPCH (Supplementary Figure 1). The GTPCH gene insert in the plasmid was confirmed using ABI 3730 sequencer (ABI, America). The plasmid construct was transfected into *Agrobacterium tumefaciens* C58C1, which was grown in MM medium containing 100 μg/mL kanamycin and rifampicin (Chen et al., 2015). Cell pellets were collected, washed, and diluted with fresh medium to a concentration of OD_600_ = 0.3 (Chen et al., 2015). The cells were incubated with an *M. alpina* CCFM 501 spore suspension and spread on cellophane membranes (Chen et al., 2015). After incubation at 23°C for 36 to 48 h in the dark, the membranes were transferred onto uracil-free synthetic complete (SC) plates supplemented with 50 μg/mL of cefotaxime and spectinomycin and incubated at 28°C until colonies appeared. The mycelia were then transferred onto uracil-free SC agar plates containing 50 μg/mL of cefotaxime and spectinomycin, followed by three consecutive subcultures to obtain stable transformants. After 8 days of cultivation in GY liquid medium (Chen et al., 2015), *M. alpina* mycelia were harvested and washed with fresh medium. Genomic DNA was extracted by the method described previously (Chen et al., 2015), and the integration of the GTPCH gene in the genome was identified by PCR using the primers HisproF1 and TrpCR1 (Supplementary Figure 1 and Table 1). The PCR conditions were as follows: denaturing at 94°C for 0.5 min, annealing at 55°C for 0.5 min, and amplification at 72°C for 1 min (25 cycles).

**TABLE 1:** Fatty acid content in different *M. alpina* strains^*a*^.

	Control	DAHP	*MA-GTPCH*	CP	*MA-GTPCH* plus CP	I-NAME	*MA-GTPCH* plus L-NAME
TFA/biomass (%, wt/wt)	30.07%	18.96%	41.24%	14.94%	24.07%	22.52%	24.95%
C16:0/biomass (%, wt/wt)	4.45%	3.34%	5.64%	2.88%	3.25%	5.21%	3.92%
C18:0/biomass (%, wt/wt)	3.36%	1.82%	5.16%	1.20%	3.19%	2.10%	3.55%
C18:1/biomass (%, wt/wt)	5.88%	5.73%	4.80%	6.14%	7.34%	6.65%	6.63%
C18:2/biomass (%, wt/wt)	2.09%	1.23%	3.00%	0.89%	1.66%	1.72%	1.75%
C18:3/biomass (%, wt/wt)	1.42%	0.93%	1.55%	0.59%	0.94%	1.34%	1.35%
C20:3/biomass (%, wt/wt)	1.26%	0.57%	1.44%	0.25%	1.13%	0.62%	0.87%
C20:4/biomass (%, wt/wt)	9.81%	4.24%	17.50%	2.50%	5.60%	4.08%	6.23%
Biomass (g/L)	11.82	11.91	11.73	11.65	11.77	11.69	11.95

*^*a*^FA (fatty acids); TFA (total fatty acids); 16:0 (palmitic acid); 18:0 (stearic acid); 18:1 (oleic acid); 18:2 (linoleic acid); 18:3 (γ-linolenic acid); 20:3 (dihomo-γ-linolenic acid); 20:4 (arachidonic acid); DAHP (2,4-diamino-6-hydroxypyrimidine); CP (4-chloro-DL-phenylalanine); L-NAME (*N*-nitro-L-arginine methyl ester); Control, *M. alpina* CCFM 501 strain containing pBIG2-ura5s-IT1 (control strain); DAHP, control strain grown on DAHP-containing medium; CP, control strain grown on CP-containing medium; *MA-GTPCH* plus CP, *MA-GTPCH* strain grown on CP-containing medium; L-NAME, control strain grown on L-NAME-containing medium; *MA-GTPCH* plus L-NAME, *MA-GTPCH* grown on L-NAME-containing medium.*

### Real-Time Quantitative PCR (qPCR)

qPCR was performed on a CFX96 qPCR System (Bio-Rad) with SYBR Green Supermix (Bio-Rad) as described previously (Wang H. et al., 2016). The amplification efficiency of each qPCR reactions was calculated based on the slope of the standard curve and a melting curve analysis was used to verify the amplification product. All the primer sets used in our experiment lie between 90 and 110% efficient and had high specificity. The ΔΔCt method was used to calculate the relative fold gene expression of samples. The control strain was used as a reference when calculating the ΔΔCt values for all the samples. The PCR conditions were as follows: 95°C for 2 min and 55°C for 5 min (40 cycles). The 18S rRNA gene was used as the reference gene. The primers are shown in Supplementary Table 1. This experiment was replicated three times.

### Determination of Biopterin and Folate Levels in *M. alpina*

BH_4_ and dihydrobiopterin (BH_2_) levels were measured by liquid chromatography and mass spectrometry (LC–MS) after iodine oxidation in acidic or alkaline conditions as described previously (Wang H. et al., 2011). Briefly, mycelial cell pellets were lysed in cold extraction buffer (50 mM Tris-HCl, pH 7.4, 1 mM DTT, 1 mM EDTA). To remove proteins, 10 μL of a 1:1 mixture of 1.5M HClO_4_ and 2M H_3_PO_4_ was added to 90 μL of extracts, followed by centrifugation. To determine total biopterins (BH_4_, BH_2_, and biopterin) by acid oxidation, 10 μL of 1% iodine in 2% KI solution was added to 90-μL supernatant. To determine BH_2_ and biopterin levels by alkali oxidation, 10 μL of 1M NaOH was added to 80 μL supernatant, followed by the addition of 10 μL iodine/KI solution. For folate extraction, the mycelia were immediately collected and frozen in liquid nitrogen and homogenized with a pestle in liquid nitrogen to extract folate. The homogenate was resuspended and mixed in 1 mL of extraction buffer (80% methanol, 0.1% ascorbic acid, and 20 mM ammonium acetate; −80°C), transferred to a 1.5 mL tube, and kept at −20°C for 15 min. The sample was then spun in a microcentrifuge at 12,000 *g* for 5 min at 4°C, and the supernatant was taken as the first extract. The pellet was then resuspended in the extraction buffer and stored at −20°C for 15 min. This sample was spun at 12,000 *g* for 15 min at 4°C, and the supernatant was taken as the second extract. The combined extract was subjected to LC–MS analysis to determine the folate level (Lu et al., 2007). This experiment was replicated three times.

### Measurement of Tyrosine, NO, and NADPH Levels in *M. alpina*

Tyrosine levels in *M. alpina* were measured as described previously (Tan et al., 2011). Briefly, 0.1 g of the mycelia was homogenized in liquid nitrogen, and the homogenate was resuspended in 1 mL of distilled water and incubated at 100°C for 30 min. A 5% trichloroacetic acid solution was added at a ratio of 1:1, and the samples were centrifuged at 12,000 *g* for 10 min at 4°C. The supernatant was filtered through a 0.45-μm cellulose syringe filter, and the obtained solution was injected into the LC–MS system. NOS activity was determined by measuring NO levels using a nitrate/nitrite assay kit (BioVision) (Wang et al., 2008). NADPH levels were measured using the NADP/NADPH quantification kit (BioVision). This experiment was replicated three times.

### Liquid Chromatography and Mass Spectrometry

Liquid chromatography and mass spectrometry was performed using a Thermo Scientific Dionex UltiMate 3000 RSLC system coupled online with the Q Exactive Hybrid Quadrupole-Orbitrap Mass Spectrometer (Thermo Fisher Scientific, United States). Chromatographic separation was performed on a TSK-Gel Amide-80 column (150 mm × 2.0 mm, 2.0 μm) maintained at 35°C. Aliquots of reaction mixtures were then injected into the column in 5 mM ammonium acetate and acetonitrile following the gradient elution program shown in Supplementary Table 2. Full scan with an m/z range of 55–700 and selected ion monitoring at m/z 440.13 (folate), m/z 238.10 (biopterin), and m/z 180.07 (tyrosine) were performed simultaneously. This experiment was replicated three times.

## Results

### BH_4_ Is of Potential Importance in Lipogenesis in *M. alpina*

**FIGURE 1:**
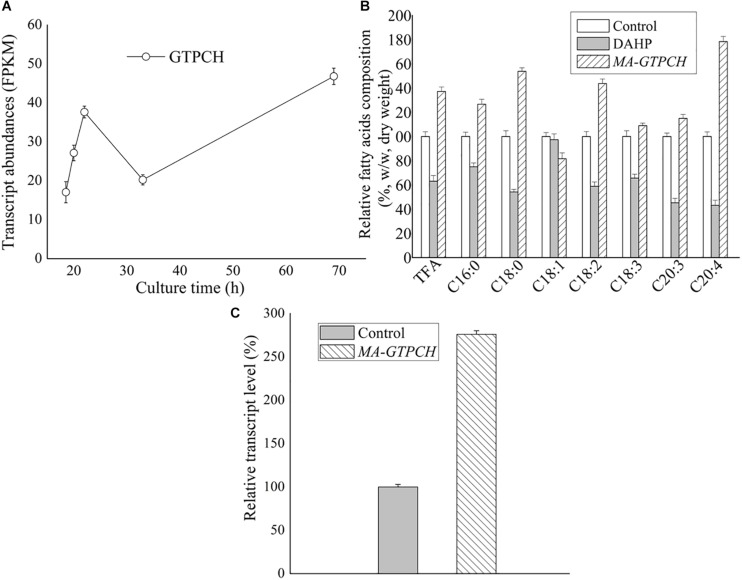
Roles of GTPCH in fatty acid biosynthesis. Transcript abundance of the GTPCH gene during lipid accumulation in wild-type *M. alpina* **(A)** (Chen et al., 2015). FPKM, Fragments per kilobase of exon per million fragments mapped. Effects of a GTPCH inhibitor and GTPCH overexpression on the fatty acid content in *M. alpina* after 8 days of cultivation **(B)**. Effects of GTPCH overexpression on the relative transcript levels of GTPCH in *M. alpina* after 8 days of cultivation **(C)**. Control, pBIG2-ura5s-IT1-containing *M. alpina* CCFM 501 strain grown on inhibitor-free medium, defined as 100%. TFA (total fatty acids); C16:0 (palmitic acid); C18:0 (stearic acid); C18:1 (oleic acid); C18:2 (linoleic acid); C18:3 (γ-linolenic acid); C20:3 (dihomo-γ-linolenic acid); C20:4 (arachidonic acid). The data shown are the averages (±standard deviations) of three independent experiments.

Previous transcriptome data were analyzed to determine the changes in the expression levels of GTPCH during the course of lipogenesis, which was induced by nitrogen exhaustion (Chen et al., 2015). Transcriptome analyses were performed using a series of samples collected when nitrogen was almost exhausted (18.5 h), just before nitrogen was used up completely (20 and 22 h), and once lipid had accumulated for a long period of time (33 and 69 h). GTPCH was transcribed at all time points (Figure 1A and Supplementary Figure 7). Notably, GTPCH expression was upregulated to almost 220% in the sample collected when lipids had begun to accumulate (22 h) compared with that in the sample collected when nitrogen was almost exhausted (18.5 h) (Figure 1A).

To explore the role of GTPCH in lipogenesis, we investigated the effects of DAHP, a GTPCH inhibitor in higher organisms, on the fatty acid content in *M. alpina* (Table 1). *M. alpina* cultures grown in a medium with DAHP showed lower total fatty acid (TFA) accumulation levels than those grown in a medium without DAHP by approximately 36% (Figure 1B), with the most pronounced change found in the levels of arachidonic acid (20:4, AA), the main commercial product of *M. alpina* (Figure 1B). In addition, the relative AA amount in *M. alpina* cultures grown in a medium with DAHP decreased by approximately 11% (from 33 to 22% of the TFA level) relative to that in *M. alpina* cultures grown in a medium without DAHP (Table 1). These findings indicate that BH_4_ is of potential importance in lipogenesis in *M. alpina*.

### Effect of GTPCH Overexpression on Intracellular Biopterin Content

The binary vector pBIG2-ura5s-GTPCH was constructed to transfer the GTPCH gene into the *M. alpina* CCFM 501 strain (uracil auxotrophic) (Hao et al., 2014). This vector harbors a T-DNA fragment (from LB to RB), which contains two expression cassettes: ura5 and GTPCH (Supplementary Figure 1). The T-DNA fragment was integrated into the chromosome of the *M. alpina* CCFM 501 strain after *A. tumefaciens*-mediated transformation. The presence of integrated T-DNA in the genomic DNA isolated from the transformed strains was confirmed by PCR using the primer pair HisproF1/TrpCR1 (Supplementary Figure 1). The presence of 818 and 971 bp products of the ura5 and GTPCH gene expression cassettes, respectively, revealed the presence of the T-DNA fragment in the genomic DNA (Supplementary Figure 2). qPCR also indicated that the transcript levels of the GTPCH gene in the strain that overexpressed GTPCH (*MA-GTPCH*) were increased significantly to approximately 276% compared with those in the *M. alpina* CCFM 501 strain that contained pBIG2-ura5s-IT1 (the control strain) (Figure 1C).

**FIGURE 2:**
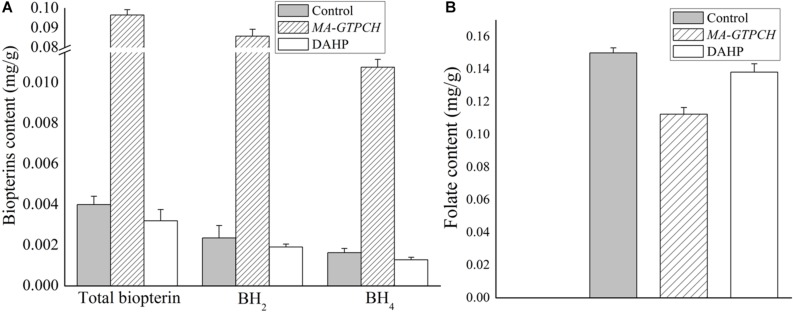
Biopterin **(A)** and folate **(B)** contents in GTPCH-overexpressing *M. alpina* strain (*MA-GTPCH*) and pBIG2-ura5s-IT1-containing *M. alpina* CCFM 501 strain (control). The data shown are the averages (±standard deviations) of three independent experiments.

Biopterin was measured to determine whether GTPCH overexpression contributed to the increase in the BH_4_ levels in *M. alpina* (Supplementary Figure 4). Total cellular biopterin, BH_4_ and BH_2_ levels in the control strain were very low (Figure 2A). In contrast, biopterin levels were 24-fold higher, BH_2_ levels were 36-fold higher, and BH_4_ levels were sevenfold higher in the *MA-GTPCH* strain due to GTPCH overexpression (Figure 2A). Furthermore, the BH_4_ levels represented 11% of the total biopterin in the *MA-GTPCH* strain compared with 41% in the control strain. However, the potential of folate biofortification was not displayed by GTPCH overexpression in the *MA-GTPCH* strain, which showed 25% lower folate content than that in the control strain (Figure 2B). Taken together, these findings show that GTPCH overexpression was highly efficient in enhancing BH_4_ levels but not folate levels in *M. alpina*.

### Effect of BH_4_ Levels on Lipogenesis

We first evaluated the fatty acid contents in *M. alpina* to determine whether increased BH_4_ levels led to increases in the lipid levels (Table 1, Supplementary Table 3 and Supplementary Figure 5). The TFA levels were 37% higher and the AA level were 78% higher in the *MA-GTPCH* strain than in the control strain (Figure 1B), and the AA level represented 42% of the TFA level in the *MA-GTPCH* strain compared with 33% of that in the control strain, suggesting increased TFA poly-unsaturation in the former (Table 1). These findings suggest that GTPCH overexpression increased both cell fatty acid contents and TFA poly-unsaturation.

### GTPCH Overexpression Promotes NADPH Generation in *M. alpina*

**FIGURE 3:**
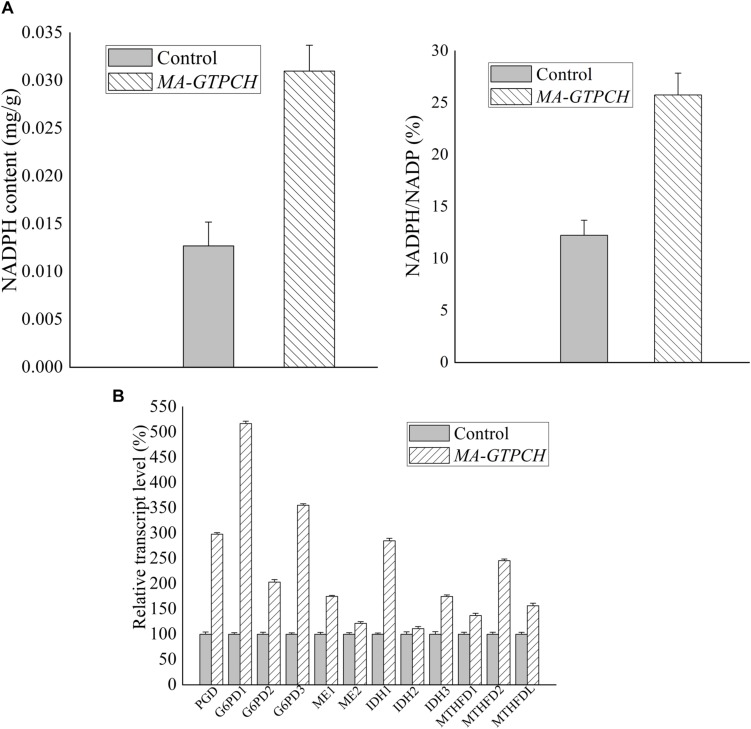
Effects of increased BH_4_ levels on the NADPH content **(A)** and transcript levels of NADPH-producing genes involved in glycolysis, PPP, phenylalanine metabolism, TCA cycle, and one carbon pool by folate in *M. alpina* **(B)**. Control, pBIG2-ura5s-IT1-containing *M. alpina* CCFM 501 strain. The data shown are the averages (±standard deviations) of three independent experiments.

To evaluate how lipid production is regulated by intracellular BH_4_ availability, the NADPH levels in the *MA-GTPCH* and control strains were measured (Supplementary Figure 6). GTPCH overexpression was found to increase NADPH production by approximately 200% (Figure 3A). To investigate NADPH regulation in the *MA-GTPCH* strain, the transcript levels of NADPH-producing genes were analyzed by qPCR in triplicate (Figure 3B).

Malic enzyme is a crucial enzyme for NADPH generation from malate (Zhang et al., 2007). GTPCH overexpression led to a 175% upregulation in ME1 expression and 121% upregulation in ME2 expression (Figure 3B). Indeed, recent studies have demonstrated that ME1 overexpression increases the fatty acid content in oleaginous microorganisms such as *M. alpina*, *Mucor circinelloides*, *Rhodotorula glutinis*, and *Yarrowia lipolytica* and that ME1 overexpression increases fatty acid unsaturation in *M. alpina* (Zhang et al., 2007; Li et al., 2013; Ratledge, 2014; Chen et al., 2015).

The upregulation of NADPH generation by glucose-6-phosphate dehydrogenase (G6PD) and 6-phosphogluconate dehydrogenase (PGD) in the PPP is one of the major determinants of efficient lipid biosynthesis in oleaginous microorganisms (Chen et al., 2015). In our study, the expression of PGD and three isoforms of G6PD was significantly upregulated (298, 517, 203, and 355%) upon GTPCH overexpression (Figure 3B). RNA interference (RNAi) of PGD and G6PD decreased the NADPH content and lipid levels in *M. alpina* (Chen et al., 2015), whereas heterologous expression of PGD and G6PD into the oleaginous yeast *Y. lipolytica* successfully increased the fatty acid level by 14%.

Although the PPP is known to be the most important pathway for NADPH production, there is a possibility that some NADPH is generated by IDH in the tricarboxylic acid (TCA) cycle (Chen et al., 2015). In our study, the expression of NADP^+^-dependent IDH (IDH1 and IDH2) was upregulated (285 and 111%) upon GTPCH overexpression (Figure 3B).

In a recent study, quantitative flux analysis of NADPH in immortalized baby mouse kidney epithelial cells revealed the crucial role of folate metabolism in NADPH generation and that most of the generated NADPH is consumed by lipogenesis (Fan et al., 2014). Although GTPCH overexpression did not increase the folate level in the *MA-GTPCH* strain in our study, the expression of NADPH-producing genes in folate metabolism, including methylenetetrahydrofolate dehydrogenase (MTHFD) 1, MTHFD2, and MTHFDL, was upregulated (137, 246, and 156%) (Figure 3B).

### Effect of Increased BH_4_ Levels on Phenylalanine Hydroxylation and NO Synthesis in *M. alpina*

In our previous study, we identified and characterized the fungal phenylalanine hydroxylation system in *M. alpina* (Wang et al., 2013). In this study, we found that GTPCH overexpression led to a 56% upregulation in PAH expression (Supplementary Figure 3A). The tyrosine content in *M. alpina* was then measured to determine whether GTPCH overexpression contributed to the enhanced phenylalanine hydroxylation. The results showed that GTPCH overexpression in *M. alpina* enhanced tyrosine production by approximately 45% (Supplementary Figure 3B), indicating that the level of phenylalanine hydroxylation was enhanced by the increased BH_4_ levels.

BH_4_ can also function as a cofactor for NOS in the fungi *Phycomyces blakesleeanus* and *Neurospora crassa*, and the NOS gene is also conserved in the *M. alpina* genome (Wang et al., 2013). In our study, we found that GTPCH overexpression upregulated the NOS expression in the *MA-GTPCH* strain by 48% (Supplementary Figure 3C). Furthermore, we measured NO levels by measuring the stable metabolite nitrite and found that the nitrite levels were significantly enhanced by approximately 32% in the *MA-GTPCH* strain compared with that in the control strain (Supplementary Figure 3D). These data indicated that electron flow in coupled NOS was diverted to arginine rather than to molecular oxygen, resulting in NO production rather than superoxide production.

### Effects of a PAH Inhibitor and an NOS Inhibitor on Fatty Acid Synthesis and NADPH Generation

**FIGURE 4:**
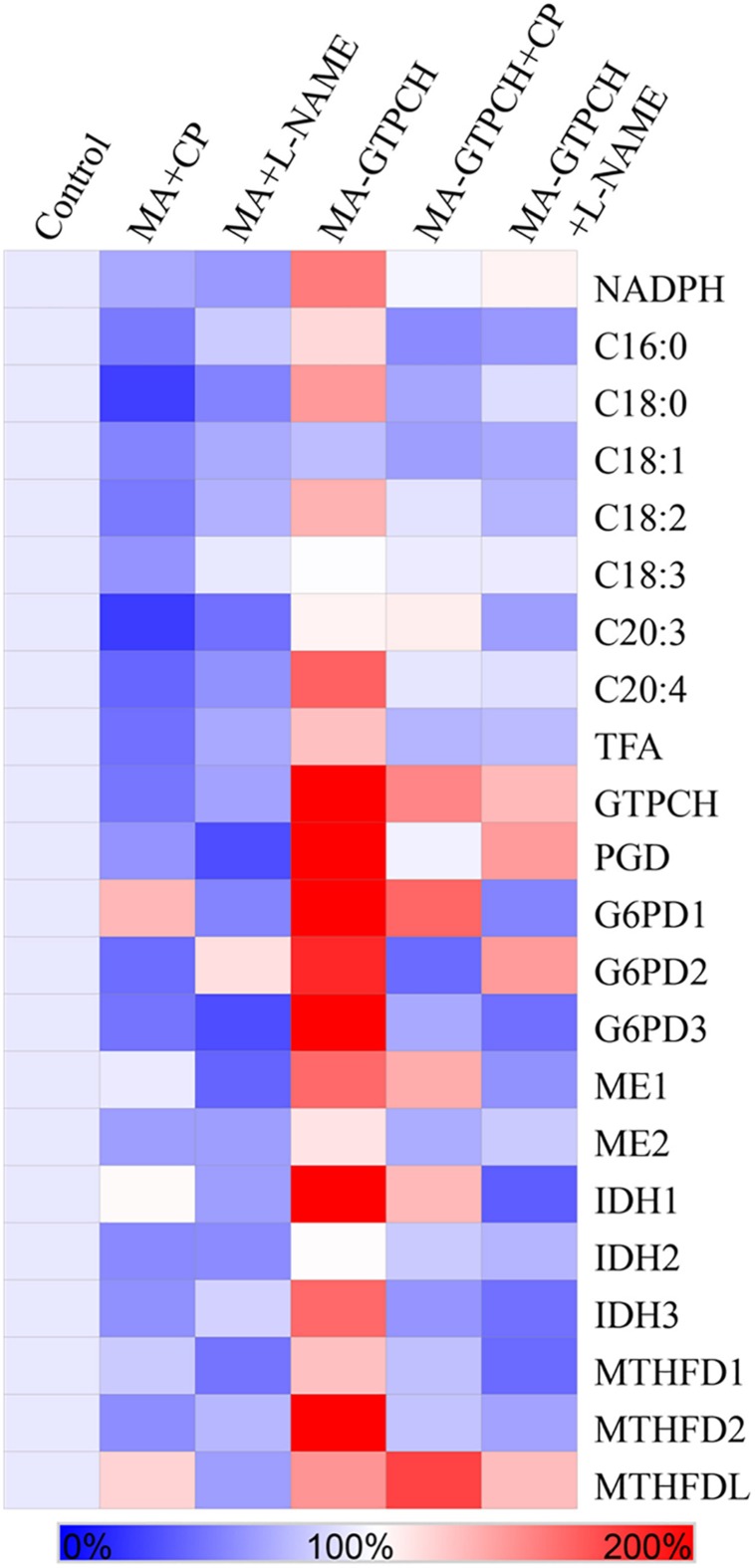
Effects of PAH and NOS inhibitors on the relative contents of NADPH and fatty acids, and relative transcript levels of NADPH-producing genes involved in glycolysis, PPP, TCA cycle, and one carbon pool by folate in the GTPCH-overexpressing *M. alpina* strain (*MA-GTPCH*) and the pBIG2-ura5s-IT1-containing *M. alpina* CCFM 501 strain (control). CP (4-chloro-DL-phenylalanine); L-NAME (*N*-nitro-L-arginine methyl ester). The data shown are the averages of three independent experiments.

To confirm the hypothesis that phenylalanine catabolism plays a key role in translating the effects of BH_4_ on lipogenesis, we investigated the effects of 5 mM 4-chloro-DL-phenylalanine (CP), a PAH inhibitor in higher organisms (Miller et al., 1976), on fatty acid synthesis in the *MA-GTPCH* and control strains (Table 1). The proenzyme form of PAH can be specifically inactivated by CP (Miller et al., 1976). Both strains grown in a medium with CP showed reduced TFA levels (Figure 4), but the TFA level in the *MA-GTPCH* cultures grown in a medium with CP was higher than that in the control strain grown in that medium. To evaluate how PAH translates the effects of BH_4_ on lipogenesis, NADPH levels in *MA-GTPCH* and *M. alpina* grown in a medium with CP were also measured (Figure 4). The addition of the PAH inhibitor in the *MA-GTPCH* and control strain cultures decreased NADPH production and downregulated the transcript levels of NADPH-producing genes, including 6PGD, G6PD, ME, IDH, and MTHFD (Figure 4), indicating that CP blocked the integrating role of phenylalanine catabolism in translating the effects of BH_4_ on lipogenesis.

To prove the hypothesis that the arginine–NO pathway may transduce the effects of BH_4_ on lipogenesis, we investigated the effects of 5 mM *N*-nitro-L-arginine methyl ester (L-NAME), an NOS inhibitor in higher organisms (Adaramoye et al., 2012), on fatty acid synthesis in the *MA-GTPCH* and control strains (Table 1). L-NAME is a structural analog of L-arginine and competes with L-arginine for NOS (Adaramoye et al., 2012). Both strains grown in a medium with L-NAME showed reduced TFA levels (Figure 4). The TFA levels in the *MA-GTPCH* cultures grown in a medium with L-NAME were higher than those in the control strain grown in that medium. To evaluate how NOS translates the effects of BH_4_ on lipid accumulation, NADPH levels in the *MA-GTPCH* and control strains grown in a medium with L-NAME were also measured (Figure 4). The results showed that the addition of the NOS inhibitor in the *MA-GTPCH* and control strain cultures decreased NADPH production and downregulated the transcript levels of NADPH-producing genes, including 6PGD, G6PD, ME, IDH, and MTHFD (Figure 4). These results indicated that L-NAME also blocked the integrating role of the arginine–NO pathway in transducing the effects of BH_4_ on lipogenesis.

## Discussion

In this study, we constructed a new fungal model of GTPCH overexpression to explore the role of BH_4_ in lipogenesis. We found that GTPCH overexpression greatly enhanced the absolute levels of BH_2_ and BH_4_ but proportionally decreased the BH_4_ fraction. The BH_2_:BH_4_ ratio tended to be high in the *MA-GTPCH* strain, suggesting a relatively insufficient recycling of BH_2_ to BH_4_ by dihydrofolate reductase (DHFR) (Wang H. C. et al., 2016). DHFR is critical for maintaining the BH_4_:BH_2_ ratio, particularly in low-biopterin conditions (Crabtree et al., 2009). Thus, the function of DHFR in BH_4_ synthesis and lipogenesis in *M. alpina* may be a fascinating topic worthy of further study. Nevertheless, our research clearly showed that GTPCH overexpression alone was capable of enhancing BH_4_ synthesis in *M. alpina*, indicating that the downstream enzymes do not play a significantly rate-limiting role in the *de novo* synthesis of BH_4_. Thus, GTPCH overexpression in *M. alpina* could provide a specific and powerful approach to explore the role of BH_4_ in lipogenesis.

It has been suggested that BH_4_ plays an important role in lipogenesis in higher organisms. Mutations in the BH_4_-dependent PAH result in the metabolic disease called phenylketonuria, the patients of which present lower concentrations of AA and eicosapentaenoic acid (Fusetti et al., 1998). In our previous research, *M. alpina* also showed reduced TFA and AA levels when grown on a medium with PAH inhibitor (Wang et al., 2013). BH_4_ in *M. alpina* was suggested to play a role in contributing NADPH for lipogenesis via phenylalanine metabolism by PAH (Wang et al., 2013). However, none of these studies were able to illuminate the functional significance of BH_4_ in lipogenesis. In our study, obvious changes were observed in the fatty acid content and status in *M. alpina* grown on a medium with GTPCH inhibitor (Figure 1B). In addition, GTPCH overexpression not only increased the cell fatty acid content but also TFA poly-unsaturation (Figure 1B). These results suggest that BH_4_ can control lipogenesis either on its own or via interactions with other as yet unrecognized regulatory factors. Collectively, our results suggest an important role of BH_4_ in lipogenesis in *M. alpina*. Because the effect of BH_4_ is probably extensive in lipogenesis, the role of BH_4_ should be deeply investigated and explored in other oleaginous microorganisms.

**FIGURE 5:**
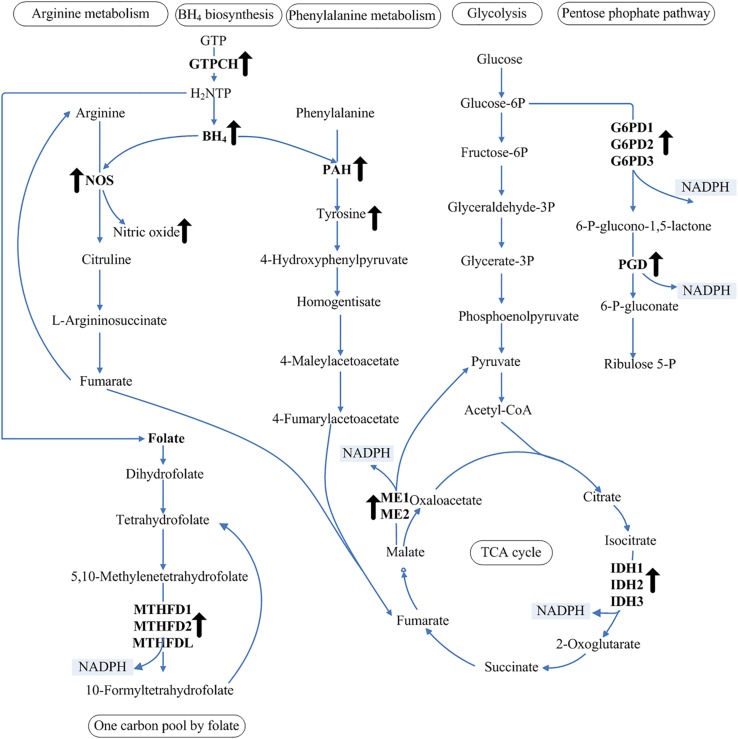
NADPH regulation in glycolysis, PPP, TCA cycle, and one carbon pool by folate. The upward arrows indicate increased levels of BH_4_, NO, and tyrosine and upregulation of transcript levels in the GTPCH-overexpressing *M. alpina* strain (*MA-GTPCH*).

NADPH is the limiting factor and a critical reducing agent in lipid biosynthesis (Wang et al., 2013). Unsaturated fatty acids are produced in the endoplasmic reticulum by the action of integral membrane-bound fatty acid desaturase enzymes that sequentially insert double bonds into the acyl chain (Michaelson et al., 1998). The Δ5-desaturase is responsible for the conversion of dihomo-γ-linolenic acid to AA using NADH or NADPH as the electron donor (Michaelson et al., 1998). Using the genomic data of *M. alpina*, the NADPH-generating pathway was constructed to map the regulatory pathway of NADPH (Figure 5). Our observation of increased NADPH levels in response to increased BH_4_ levels in the *MA-GTPCH* strain suggests a novel aspect of intracellular NADPH regulation by BH_4_. Compared with the samples from the control strain, those from the *MA-GTPCH* strain showed upregulated expression of all NADPH-producing genes when the lipid had accumulated for a long period of time. Thus, BH_4_ likely promotes an elegant coordination of glycolysis, PPP, TCA cycle, and one carbon pool by folate to enhance lipid synthesis and poly-unsaturation via NADPH regulation in *M. alpina* (Figure 5).

The intracellular BH_4_ level was significantly increased upon GTPCH overexpression, which enhanced the function of phenylalanine catabolism and the arginine–NO pathway. The addition of an NOS or a PAH inhibitor in the *MA-GTPCH* and control strain cultures decreased fatty acid accumulation, NADPH production, and the transcript levels of NADPH-producing genes, which confirmed the transduction effects of phenylalanine catabolism and arginine–NO pathway (Figure 4). The metabolism of amino acids such as leucine, lysine, or gamma-amino butyric acid has been reported to be another limiting factor in lipogenesis in oleaginous fungi (Kamisaka et al., 2007; Rodriguez-Frometa et al., 2012; Vorapreeda et al., 2012; Liu et al., 2015). Fumarate is the end product of both phenylalanine catabolism and the arginine–NO pathway (Figure 5), which could potentiate ME activity by binding to separate allosteric sites (Yang et al., 2002; Hsieh et al., 2014). This information suggests that the enhancement of phenylalanine hydroxylation and NO synthesis could lead to significant changes in carbon flux and result in an imbalance of the TCA cycle intermediates, further allowing more flux via lipogenesis (Figure 5).

BH_4_ is also a major determinant of whether NOS produces NO or superoxide and thus plays a key role in regulating cellular oxidative stress (Schulz et al., 2008). *In vivo* studies have demonstrated that increased BH_4_ levels could enhance the NO content and inhibit vascular oxidative stress in transgenic mouse models of GTPCH overexpression (Alp et al., 2004; Tie et al., 2009). In our study, increased BH_4_ level enhanced the function of NOS in the *MA-GTPCH* strain, resulting in the generation of NO rather than superoxide, thus suppressing oxidative stress (Figure 5) (Schulz et al., 2008). Oxidative stress has recently emerged as a key regulatory factor for lipogenesis, which can attenuate lipid synthesis, decrease fatty acid chain length and unsaturation, and increase fatty acid oxidation (Assies et al., 2014; Douglas et al., 2016). The contributions of NADP^+^-reducing enzymes to the NADPH pool has been shown to be altered by oxidative stress (Rzezniczak and Merritt, 2012). Enhancement of NO synthesis in *M. alpina* may also increase the contributions of NADP^+^-reducing enzymes to the NADPH pool by suppressing oxidative stress (Figure 5). In higher organisms, the biological actions of NO are mainly mediated by the direct activation of soluble guanylyl cyclase and the consequent increase in intracellular cGMP levels, and the NO/cGMP signaling pathway is endowed with regulatory properties in fatty acid metabolism (GarcıA-Villafranca et al., 2003). NO can also lead to the activation of sterol regulatory element-binding proteins, which may play important roles in lipid accumulation (Gharavi et al., 2006).

## Conclusion

Our study established the utility of GTPCH overexpression as a specific and novel approach to enhance intracellular BH_4_ levels and revealed that the availability of BH_4_ is critical for lipogenesis in *M. alpina*. Phenylalanine catabolism and NO synthesis may play an integrating role in translating the effects of BH_4_ on lipogenesis by regulating the intracellular NAPDH pool. RNAi of the PAH or NOS gene should be adopted in future studies to explore the mechanisms of BH_4_-induced lipogenesis. Our findings provide novel insights into the mechanisms of efficient lipid biosynthesis regulation in oleaginous microorganisms and lay a foundation for the genetic engineering of these organisms to optimize the production of dietary fat.

## Data Availability Statement

The raw data supporting the conclusions of this article will be made available by the authors, without undue reservation, to any qualified researcher.

## Author Contributions

All authors contributed to the conception and planning of the study. HW and CZ performed the experiments and drafted the manuscript. HC, JZ, WC, and ZG supervised the experimental work. HZ, YC, and WC reviewed the manuscript. All authors read and approved the final manuscript.

## Conflict of Interest

The authors declare that the research was conducted in the absence of any commercial or financial relationships that could be construed as a potential conflict of interest.

## Footnotes

1 http://www.ncbi.nlm.nih.gov/Traces/sra/

## References

[B1] Adaramoye O. A. Nwosu I. O. Farombi E. O. (2012). Sub-acute effect of N(G)-nitro-l-arginine methyl-ester (L-NAME) on biochemical indices in rats: protective effects of kolaviron and extract of Curcuma longa L. *Pharmacognosy Res.* 4 127–133. 10.4103/0974-8490.99071 22923949 PMC3424838

[B2] Alp N. J. McAteer M. A. Khoo J. Choudhury R. P. Channon K. M. (2004). Increased endothelial tetrahydrobiopterin synthesis by targeted transgenic GTP-cyclohydrolase I overexpression reduces endothelial dysfunction and atherosclerosis in ApoE-knockout mice. *Arterioscler. Thromb. Vasc. Biol.* 24 445–450. 10.1161/01.ATV.0000115637.48689.77 14707037

[B3] Assies J. Mocking R. J. Lok A. Ruhe H. G. Pouwer F. Schene A. H. (2014). Effects of oxidative stress on fatty acid- and one-carbon-metabolism in psychiatric and cardiovascular disease comorbidity. *Acta Psychiatr. Scand.* 130 163–180. 10.1111/a.12265 24649967 PMC4171779

[B4] Chaney A. L. Marbach E. P. (1962). Modified reagents for determination of urea and ammonia. *Clin. Chem.* 8 130–132. 10.1016/0009-8981(62)90080-3 13878063

[B5] Chen H. Hao G. Wang L. Wang H. Gu Z. Liu L. (2015). Identification of a critical determinant that enables efficient fatty acid synthesis in oleaginous fungi. *Sci. Rep.* 5:11247. 10.1038/srep11247 26059272 PMC4462047

[B6] Crabtree M. J. Tatham A. L. Hale A. B. Alp N. J. Channon K. M. (2009). Critical role for tetrahydrobiopterin recycling by dihydrofolate reductase in regulation of endothelial nitric-oxide synthase coupling: relative importance of the de novo biopterin synthesis versus salvage pathways. *J. Biol. Chem.* 284 28128–28136. 10.1074/jbc.M109.041483 19666465 PMC2788863

[B7] Douglas D. N. Pu C. H. Lewis J. T. Bhat R. Anwar-Mohamed A. Logan M. (2016). Oxidative stress attenuates lipid synthesis and increases mitochondrial fatty acid oxidation in hepatoma cells infected with hepatitis C virus. *J. Biol. Chem.* 291 1974–1990. 10.1074/jbc.M115.674861 26627833 PMC4722472

[B8] Dourou M. Aggeli D. Papanikolaou S. Aggelis G. (2018). Critical steps in carbon metabolism affecting lipid accumulation and their regulation in oleaginous microorganisms. *Appl. Microbiol. Biotechnol.* 102 2509–2523. 10.1007/s00253-018-8813-z 29423634

[B9] Du Y. H. Guan Y. Y. Alp N. J. Channon K. M. Chen A. F. (2008). Endothelium-specific GTP cyclohydrolase I overexpression attenuates blood pressure progression in salt-sensitive low-renin hypertension. *Circulation* 117 1045–1054. 10.1161/CIRCULATIONAHA.107.748236 18268143

[B10] Fan J. Ye J. Kamphorst J. J. Shlomi T. Thompson C. B. Rabinowitz J. D. (2014). Quantitative flux analysis reveals folate-dependent NADPH production. *Nature* 510 298–302. 10.1038/nature13236 24805240 PMC4104482

[B11] Forrest H. S. Van Baalen C. (1970). Microbiology of unconjugated pteridines. *Annu. Rev. Microbiol.* 24 91–108. 10.1146/annurev.mi.24.100170.000515 4927141

[B12] Fusetti F. Erlandsen H. Flatmark T. Stevens R. C. (1998). Structure of tetrameric human phenylalanine hydroxylase and its implications for phenylketonuria. *J. Biol. Chem.* 273 16962–16967. 10.1074/jbc.273.27.16962 9642259

[B13] GarcıA-Villafranca J. Guillén A. Castro J. (2003). Involvement of nitric oxide/cyclic GMP signaling pathway in the regulation of fatty acid metabolism in rat hepatocytes. *Biochem. Pharmacol.* 65 807–812. 10.1016/s0006-2952(02)01623-4 12632570

[B14] Ge Z. D. Ionova I. A. Vladic N. Pravdic D. Hirata N. Vasquez-Vivar J. (2011). Cardiac-specific overexpression of GTP cyclohydrolase 1 restores ischaemic preconditioning during hyperglycaemia. *Cardiovasc. Res.* 91 340–349. 10.1093/cvr/cvr079 21422102 PMC3125073

[B15] Gharavi N. M. Baker N. A. Mouillesseaux K. P. Yeung W. Honda H. M. Hsieh X. (2006). Role of endothelial nitric oxide synthase in the regulation of SREBP activation by oxidized phospholipids. *Circ. Res.* 98 768–776. 10.1161/01.res.0000215343.89308.93 16497987

[B16] Giovannini M. Biasucci G. Agostoni C. Luotti D. Riva E. (1995). Lipid status and fatty acid metabolism in phenylketonuria. *J. Inherit. Metab. Dis.* 18 265–272. 10.1007/BF00710414 7474891

[B17] Hao G. Chen H. Wang L. Gu Z. Song Y. Zhang H. (2014). Role of malic enzyme during fatty acid synthesis in the oleaginous fungus *Mortierella alpina*. *Appl. Environ. Microbiol.* 80 2672–2678. 10.1128/AEM.00140-14 24532075 PMC3993310

[B18] Hsieh J. Y. Li S. Y. Chen M. C. Yang P. C. Chen H. Y. Chan N. L. (2014). Structural characteristics of the nonallosteric human cytosolic malic enzyme. *Biochim. Biophys. Acta* 1844 1773–1783. 10.1016/j.bbapap.2014.06.019 24998673

[B19] Ji X. J. Huang H. (2018). Engineering microbes to produce polyunsaturated fatty acids. *Trends Biotechnol.* 37 344–346. 10.1016/j.tibtech.2018.10.002 30376959

[B20] Ji X. J. Ren L. J. Nie Z. K. Huang H. Ouyang P. K. (2014). Fungal arachidonic acid-rich oil: research, development and industrialization. *Crit. Rev. Biotechnol.* 34 197–214. 10.3109/07388551.2013.778229 23631634

[B21] Kamisaka Y. Tomita N. Kimura K. Kainou K. Uemura H. (2007). DGA1 (diacylglycerol acyltransferase gene) overexpression and leucine biosynthesis significantly increase lipid accumulation in the Deltasnf2 disruptant of *Saccharomyces cerevisiae*. *Biochem. J.* 408 61–68. 10.1042/BJ20070449 17688423 PMC2049070

[B22] Kappock T. J. Caradonna J. P. (1996). Pterin-dependent amino acid hydroxylases. *Chem. Rev.* 96 2659–2756. 10.1021/cr9402034 11848840

[B23] Kaufman S. (1967). Pteridine cofactors. *Annu. Rev. Biochem.* 36 171–184. 10.1146/annurev.bi.36.070167.001131 18257719

[B24] Kaufman S. (1993). New tetrahydrobiopterin-dependent systems. *Annu. Rev. Nutr.* 13 261–286. 10.1146/annurev.nu.13.070193.001401 8103664

[B25] Kolinsky M. A. Gross S. S. (2004). The mechanism of potent GTP cyclohydrolase I inhibition by 2,4-diamino-6-hydroxypyrimidine: requirement of the GTP cyclohydrolase I feedback regulatory protein. *J. Biol. Chem.* 279 40677–40682. 10.1074/jbc.M405370200 15292175

[B26] Li Z. Sun H. Mo X. Li X. Xu B. Tian P. (2013). Overexpression of malic enzyme (ME) of *Mucor circinelloides* improved lipid accumulation in engineered *Rhodotorula glutinis*. *Appl. Microbiol. Biotechnol.* 97 4927–4936. 10.1007/s00253-012-4571-5 23179623

[B27] Liu L. Pan A. Spofford C. Zhou N. Alper H. S. (2015). An evolutionary metabolic engineering approach for enhancing lipogenesis in *Yarrowia lipolytica*. *Metab. Eng.* 29 36–45. 10.1016/j.ymben.2015.02.003 25724340

[B28] Lu W. Kwon Y. K. Rabinowitz J. D. (2007). Isotope ratio-based profiling of microbial folates. *J. Am. Soc. Mass Spectrom.* 18 898–909. 10.1016/j.jasms.2007.01.017 17360194 PMC1909916

[B29] Michaelson L. V. Lazarus C. M. Griffiths G. Napier J. A. Stobart A. K. (1998). Isolation of a delta5-fatty acid desaturase gene from *Mortierella alpina*. *J. Biol. Chem.* 273 19055–19059. 10.1074/jbc.273.30.19055 9668087

[B30] Miller M. R. McClure D. Shiman R. (1976). Mechanism of inactivation of phenylalanine hydroxylase by p-chlorophenylalanine in hepatome cells in culture. Two possible models. *J. Biol. Chem.* 251 3677–3684. 10.1016/0014-5793(70)80512-9 180005

[B31] Mortazavi A. Williams B. A. McCue K. Schaeffer L. Wold B. (2008). Mapping and quantifying mammalian transcriptomes by RNA-Seq. *Nat. Methods* 5 621–628. 10.1038/nmeth.1226 18516045 PMC13303166

[B32] Moseley K. Koch R. Moser A. B. (2002). Lipid status and long-chain polyunsaturated fatty acid concentrations in adults and adolescents with phenylketonuria on phenylalanine-restricted diet. *J. Inherit. Metab. Dis.* 25 56–64. 10.1023/a:1015142001578 11999981

[B33] Neckameyer W. S. Coleman C. M. Eadie S. Goodwin S. F. (2007). Compartmentalization of neuronal and peripheral serotonin synthesis in *Drosophila melanogaster*. *Genes Brain Behav.* 6 756–769. 10.1111/j.1601-183X.2007.00307.x 17376153

[B34] Ratledge C. (2014). The role of malic enzyme as the provider of NADPH in oleaginous microorganisms: a reappraisal and unsolved problems. *Biotechnol. Lett.* 36 1557–1568. 10.1007/s10529-014-1532-3 24752812

[B35] Rodriguez-Frometa R. A. Gutierrez A. Torres-Martinez S. Garre V. (2012). Malic enzyme activity is not the only bottleneck for lipid accumulation in the oleaginous fungus *Mucor circinelloides*. *Appl. Microbiol. Biotechnol.* 97 3063–3072. 10.1007/s00253-012-4432-2 23053085

[B36] Rudzite V. Jurika E. Baier-Bitterlich G. Widner B. Reibnegger G. Fuchs D. (1998). Pteridines and lipid metabolism. *Pteridines* 9 103–112. 10.1515/pteridines.1998.9.2.103

[B37] Rzezniczak T. Z. Merritt T. J. (2012). Interactions of NADP-reducing enzymes across varying environmental conditions: a model of biological complexity. *G3 (Bethesda)* 2 1613–1623. 10.1534/g3.112.003715 23275884 PMC3516483

[B38] Sakuradani E. Abe T. Iguchi K. Shimizu S. (2005). A novel fungal omega3-desaturase with wide substrate specificity from arachidonic acid-producing *Mortierella alpina* 1S-4. *Appl. Microbiol. Biotechnol.* 66 648–654. 10.1007/s00253-004-1760-x 15538555

[B39] Sakuradani E. Kobayashi M. Ashikari T. Shimizu S. (1999a). Identification of delta12-fatty acid desaturase from arachidonic acid-producing mortierella fungus by heterologous expression in the yeast *Saccharomyces cerevisiae* and the fungus *Aspergillus oryzae*. *Eur. J.Biochem.* 261 812–820. 10.1046/j.1432-1327.1999.00333.x 10215899

[B40] Sakuradani E. Kobayashi M. Shimizu S. (1999b). Delta6-fatty acid desaturase from an arachidonic acid-producing *Mortierella* fungus. Gene cloning and its heterologous expression in a fungus, *Aspergillus*. *Gene* 238 445–453. 10.1016/s0378-1119(99)00359-5 10570972

[B41] Sakuradani E. Kobayashi M. Shimizu S. (1999c). Delta 9-fatty acid desaturase from arachidonic acid-producing fungus. Unique gene sequence and its heterologous expression in a fungus, *Aspergillus*. *Eur. J. Biochem.* 260 208–216. 10.1046/j.1432-1327.1999.00131.x 10091601

[B42] Schulz E. Jansen T. Wenzel P. Daiber A. Munzel T. (2008). Nitric oxide, tetrahydrobiopterin, oxidative stress, and endothelial dysfunction in hypertension. *Antioxid. Redox Signal.* 10 1115–1126. 10.1089/ars.2007.1989 18321209

[B43] Tan F. Tan C. Zhao A. Li M. (2011). Simultaneous determination of free amino acid content in tea infusions by using high-performance liquid chromatography with fluorescence detection coupled with alternating penalty trilinear decomposition algorithm. *J. Agric. Food Chem.* 59 10839–10847. 10.1021/jf2023325 21894956

[B44] Tie L. Li X. J. Wang X. Channon K. M. Chen A. F. (2009). Endothelium-specific GTP cyclohydrolase I overexpression accelerates refractory wound healing by suppressing oxidative stress in diabetes. *Am. J. Physiol. Endocrinol. Metab.* 296 1423–1429. 10.1152/ajpendo.00150.2009 19336662 PMC2692395

[B45] Trapnell C. Williams B. A. Pertea G. Mortazavi A. Kwan G. van Baren M. J. (2010). Transcript assembly and quantification by RNA-Seq reveals unannotated transcripts and isoform switching during cell differentiation. *Nat. Biotechnol.* 28 511–515. 10.1038/nbt.1621 20436464 PMC3146043

[B46] Vorapreeda T. Thammarongtham C. Cheevadhanarak S. Laoteng K. (2012). Alternative routes of acetyl-CoA synthesis identified by comparative genomic analysis: involvement in the lipid production of oleaginous yeast and fungi. *Microbiology* 158(Pt 1) 217–228. 10.1099/mic.0.051946-0 22016567

[B47] Wang H. Chen H. Hao G. Yang B. Feng Y. Wang Y. (2013). Role of the phenylalanine-hydroxylating system in aromatic substance degradation and lipid metabolism in the oleaginous fungus *Mortierella alpina*. *Appl. Environ. Microbiol.* 79 3225–3233. 10.1128/AEM.00238-13 23503309 PMC3685260

[B48] Wang H. Yang B. Hao G. Feng Y. Chen H. Feng L. (2011). Biochemical characterization of the tetrahydrobiopterin synthesis pathway in the oleaginous fungus *Mortierella alpina*. *Microbiology* 157 3059–3070. 10.1099/mic.0.051847-0 21852350 PMC4811656

[B49] Wang H. Zhang C. Chen H. Yang Q. Zhou X. Gu Z. (2016). Characterization of fungal l-fucokinase involved in *Mortierella alpina* GDP-L-fucose salvage pathway. *Glycobiology* 26 880–887. 10.1093/glycob/cww032 26957583

[B50] Wang H. C. Zhang C. Feng J. H. Liu Y. Yang Q. Chen H. Q. (2016). Role of dihydrofolate reductase in tetrahydrobiopterin biosynthesis and lipid metabolism in the oleaginous fungus *Mortierella alpina*. *Microbiology* 162 1544–1553. 10.1099/mic.0.000345 27488762

[B51] Wang L. Chen W. Feng Y. Ren Y. Gu Z. Chen H. (2011). Genome characterization of the oleaginous fungus *Mortierella alpina*. *PLoS One* 6:e28319. 10.1371/journal.pone.0028319 22174787 PMC3234268

[B52] Wang W. Zolty E. Falk S. Summer S. Zhou Z. Gengaro P. (2008). Endotoxemia-related acute kidney injury in transgenic mice with endothelial overexpression of GTP cyclohydrolase-1. *Am. J. Physiol. Renal Physiol.* 294 571–576. 10.1152/ajprenal.00538.2007 18171994

[B53] Yang Z. Lanks C. W. Tong L. (2002). Molecular mechanism for the regulation of human mitochondrial NAD(P)+-dependent malic enzyme by ATP and fumarate. *Structure* 10 951–960. 10.1016/S0969-2126(02)00788-8 12121650

[B54] Zhang Y. Adams I. P. Ratledge C. (2007). Malic enzyme: the controlling activity for lipid production? Overexpression of malic enzyme in *Mucor circinelloides* leads to a 2.5-fold increase in lipid accumulation. *Microbiology* 153 2013–2025. 10.1099/mic.0.2006/002683-0 17600047

