# Supplementary material for: Tetrahydrobiopterin Plays a Functionally Significant Role in Lipogenesis in the Oleaginous Fungus Mortierella alpina
Source: Front Microbiol. 2020 Feb 20;11:250. doi: 10.3389/fmicb.2020.00250 (PMC7044132; doi:10.3389/fmicb.2020.00250)
Supplement: Supplementary file 1 [file Data_Sheet_1.docx]

Supplementary Material

# Supplementary Figures and Tables

## Supplementary Figures


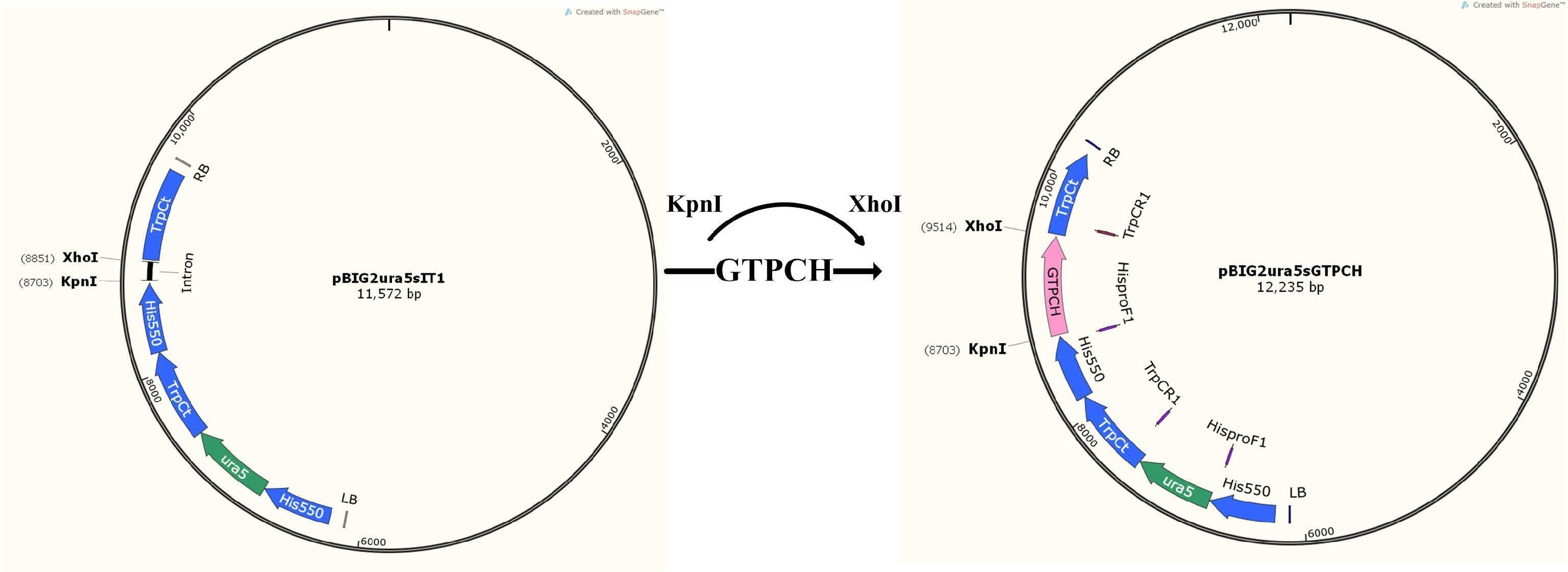


**Supplementary Figure 1.** The construction of recombinant plasmid pBIG2ura5sGTPCH. The integration of GTPCH in the genome was identified by PCR using primers HisproF1 and TrpCR1. HisproF1 and TrpCR1 can amplify two fragments, one is ura5 expression cassette, the other is GTPCH overexpression cassette. The expression of ura5 gene is the selective marker which is used for the characterization of the GTPCH transformants. RB, right border; LB, left border; GTPCH, GTP cyclohydrolase I; ura5, orotate phosphoribosyl transferase.


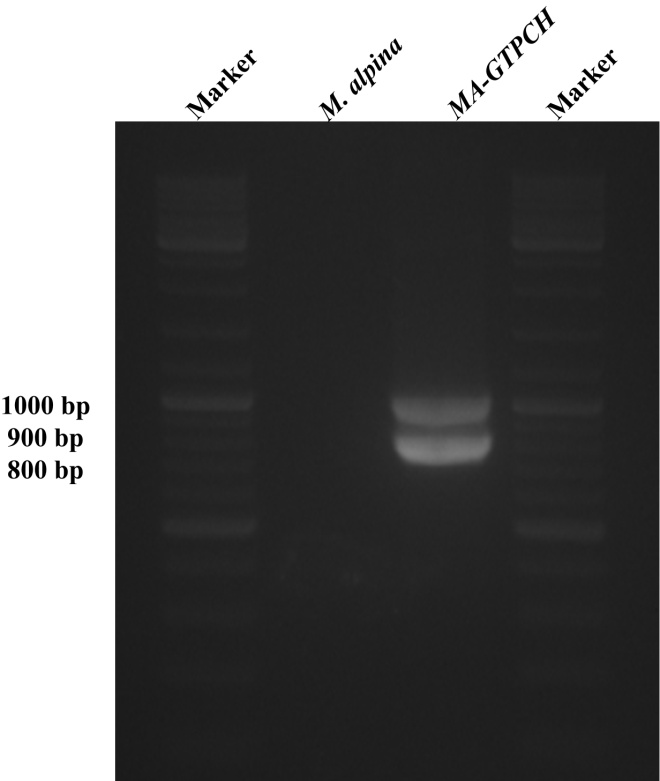


**Supplementary Figure 2.** The agarose gel electrophoresis of the PCR products from GTPCH over-expressing strain (*MA-GTPCH*). Transformation was checked by PCR using primers: HisproF1 and TrpCR1.


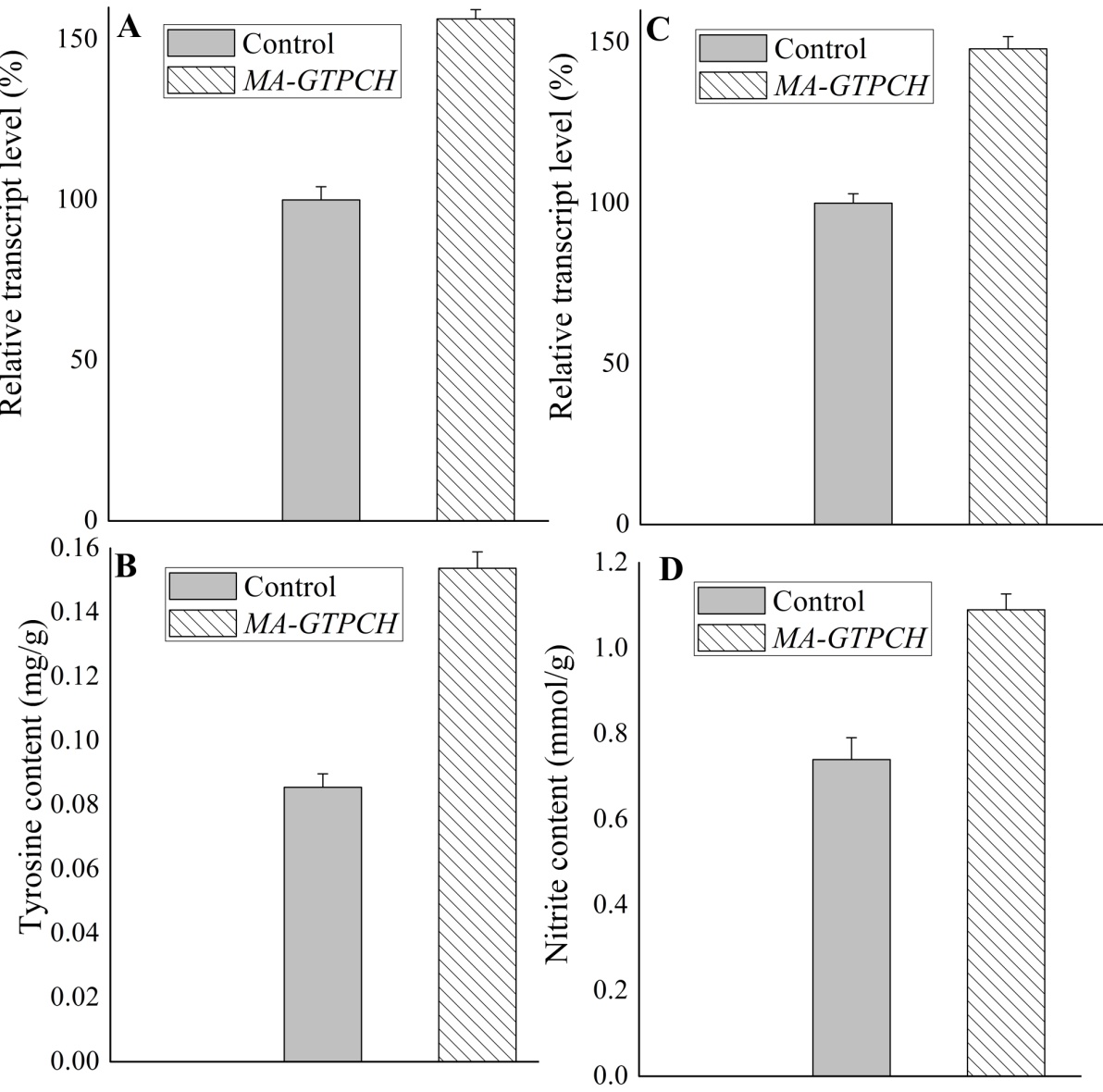


**Supplementary Figure 3.** Effects of increased BH4 levels on phenylalanine hydroxylation and nitric oxide synthesis in *M. alpina*. Transcript levels of PAH (A) and NOS (B) genes in GTPCH-overexpressing strain (*MA-GTPCH*) and *M. alpina* CCFM 501 strains with pBIG2ura5sIT1 (control strains). Tyrosine (C) and nitrite (D) content in *MA-GTPCH* and control strains. The data shown is the average (±standard deviation) of three independent experiments.


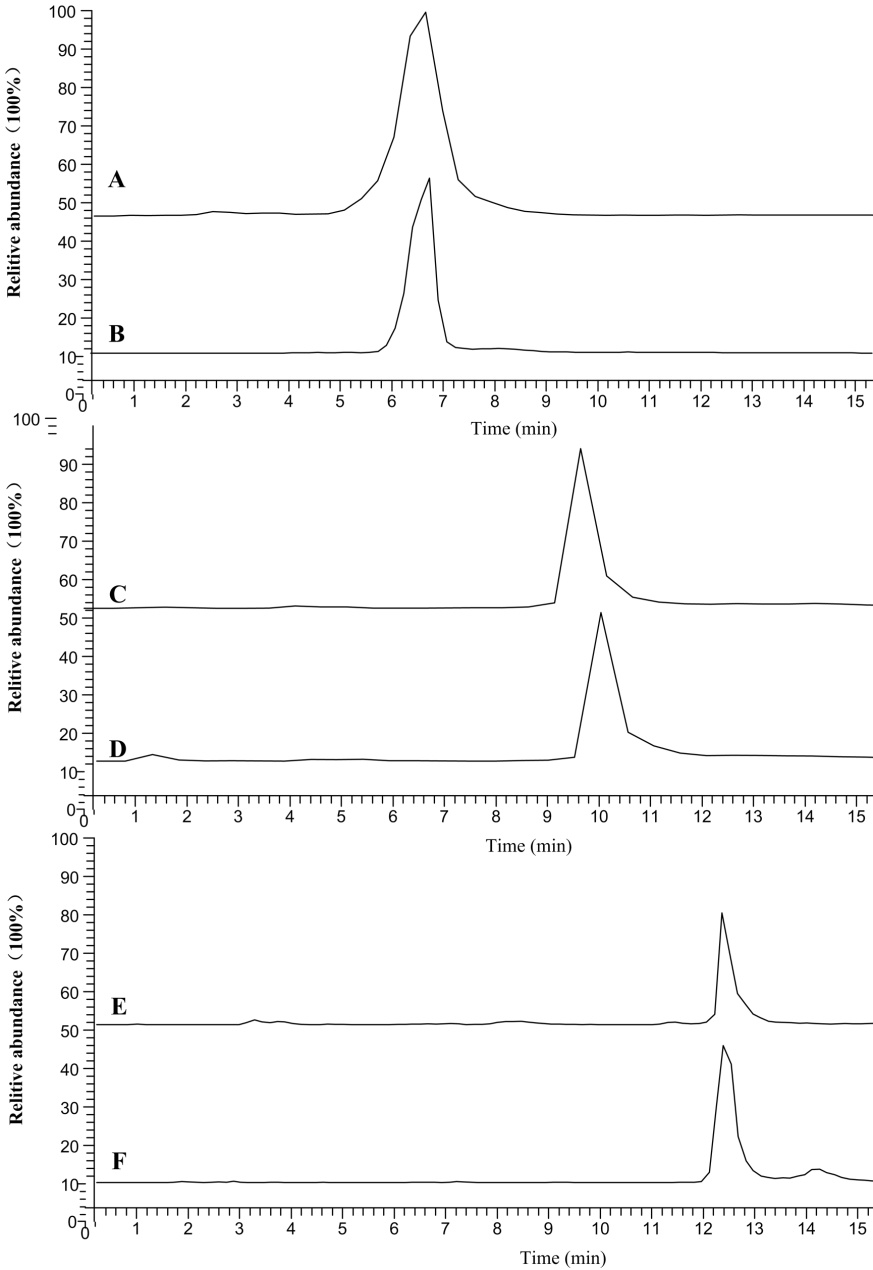


**Supplementary Figure 4.** Liquid chromatography and mass spectrometry chromatographs: (A) Extracted ion chromatogram (XIC) of the biopterin standard (m/z 238.10); (B) XIC of the biopterin determination (m/z 238.10); (C) XIC of the folate standard (m/z 440.13); (D) XIC of the folate determination (m/z 440.13); (E) XIC of the tyrosine standard (m/z 180.07); (F) XIC of the tyrosine determination (m/z 180.07).

**
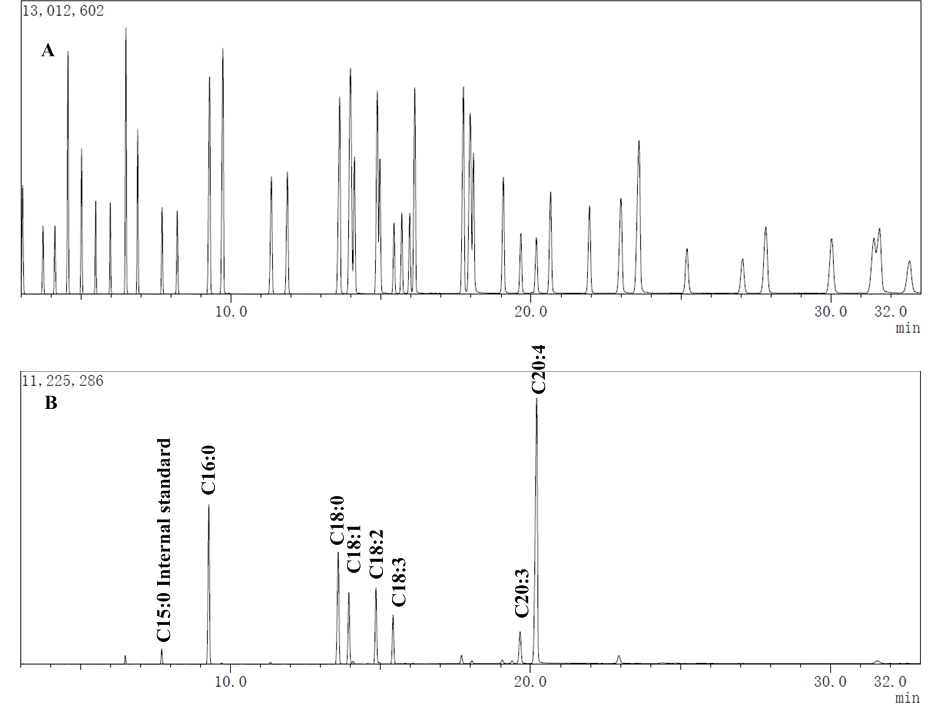
**

**Supplementary Figure 5.** Gas chromatography and mass spectrometry chromatographs: (A) 37-component fatty acids methyl ester mix; (B) fatty acids methyl ester from *M. alpina*.

**Supplementary Figure 6.** Standard curve of NADPH quantification.


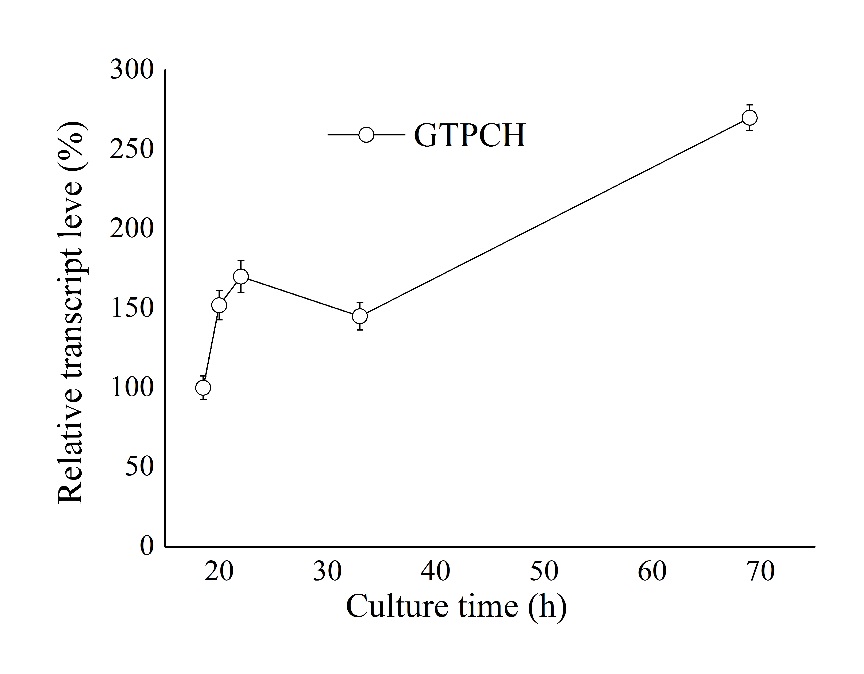


**Supplementary Figure 7.** Relative transcript level the GTPCH gene during lipid accumulation in wild-type *M. alpina*.

## Supplementary Tables

**Supplementary Table 1.** Primers used in this study.

| *Gene* | *Primer sequence^a^* |
| --- | --- |
| GTPCH-F | CGGGGTACCGCATGGCAAGCAGCATCGAGAAC |
| GTPCH-R | CCGCTCGAGAACTGCCTCTGCAATCCTCTCGATC |
| HisproF1 | CACACACAAACCTCTCTCCCACT |
| TrpCR1 | CAAATGAACGTATCTTATCGAGATCC |
| GTPCH-F (qPCR) | GACGGCCTTAGCTTCCCAAG |
| GTPCH-R (qPCR) | CGAACTGCCTCTGCAATCCT |
| PGD-F (qPCR) | AAGTTGCCTGTCCGCCATC |
| PGD-R (qPCR) | TAGTGCCAGCCGTTCTCCTT |
| G6PD1-F (qPCR) | TGGCTATCCCGCCTATTGC |
| G6PD1-R (qPCR) | TTTCGTGCTGCCTTTGGG |
| G6PD2-F (qPCR) | CCTAAGGACTGGTGCCTGTTG |
| G6PD2-R (qPCR) | CTTGGCTTGCTGTCTGCGT |
| G6PD3-F (qPCR) | CGTATGCTGGGTCTGGTTAGG |
| G6PD3 -R (qPCR) | AGAAGGCTAGGTCTCCCGATG |
| ME1-F (qPCR) | GGCTGTTGCCGAAGGGACT |
| ME1-R (qPCR) | GGCAAAGGTGGTGCTGATTTC |
| ME2-F (qPCR) | CCTTGCAGGACCGTAACGAGA |
| ME2-R (qPCR) | CCTGGAGCGACGATAAATGGA |
| IDH1-F (qPCR) | CGCCCAGATGCTCAAGTCCT |
| IDH1 -R (qPCR) | CCATCGGGCGTCAACAGAA |
| IDH2 -F (qPCR) | CCCCAAGACGGAGACAGGAC |
| IDH2 -R (qPCR) | ACAGGCGGCACAACGGATA |
| IDH3 -F (qPCR) | CTCGTCCCTGGGTGGACAG |
| IDH3-R (qPCR) | CCATCAGCGGGCGTAAAA |
| MTHFD1 -F (qPCR) | TCCTGGTAGTCGCAATCGG |
| MTHFD1-R (qPCR) | GCGGCGCTATACTCAACGT |
| MTHFD2 -F (qPCR) | GCAAGCAGGCTGGGATT |
| MTHFD2-R (qPCR) | CAAAGTCAGGCATGGTCGA |
| MTHFDL-F (qPCR) | GTTTACAACGGCATCCTTCCC |
| MTHFDL-R (qPCR) | CCTCAACCTTCAGCGTCGTCT |
| PAH-F (qPCR) | CCGCCGATTGGGACTATGA |
| PAH-R(qPCR) | CCATCTTGCGAGGGAACCAT |
| NOS-F (qPCR) | CGGACGTACAGGAGCAACC |
| NOS-R (qPCR) | AGGAAAGAGGACGGAGGGT |
| 18S rDNA-F | CTATTGGCGGAGGTCTATTCGT |
| 18S rDNA-R | GCACGCATTCGGATAATTGGT |

^a^ Restriction cleavage sites are underlined. GTPCH-F/R, primers used for amplification of GTPCH gene; HisproF1/TrpCR1, primers used for the confirmation of ura5 gene and GTPCH gene in the genome; 18S rDNA-F/R, primers used for qPCR analysis of housekeeping gene; other primers used for the detection of transcript levels with qPCR.

**Supplementary Table 2.** Gradient elution of the chromatographic separation

| Time  (min) | Flow rate  (mL/min) | B% |
| --- | --- | --- |
| 0 | 0.2 | 80 |
| 2 | 0.2 | 80 |
| 6 | 0.2 | 55 |
| 10 | 0.2 | 55 |
| 11 | 0.2 | 40 |
| 15 | 0.2 | 40 |
| 16 | 0.2 | 80 |
| 20 | 0.2 | 80 |

**Supplementary Table 3.** The status of fatty acids in different *M. alpina* strains^a^. ^a^FA (fatty acids); TFA (total fatty acids); 16:0 (palmitic acid); 18:0 (stearic acid); 18:1 (oleic acid); 18:2 (linoleic acid); 18:3 (ɤ-linolenic acid); 20:3 (dihomo-ɤ-linolenic acid); 20:4 (arachidonic acid); DAHP (2,4-diamino-6-hydroxypyrimidine); CP (4-chloro-DL-phenylalanine); L-NAME (N-Nitro-L-Arginine Methyl Ester); Control, *M. alpina* CCFM 501 strains with pBIG2ura5sIT1 (control strain); DAHP, control strain grown on DAHP medium; CP, control strain grown on CP medium; *MA-GTPCH* plus CP, *MA-GTPCH* grown on CP medium; L-NAME, control strain grown on L-NAME medium; *MA-GTPCH* plus L-NAME, *MA-GTPCH* grown on L-Name medium.

| FA/TFA (%, wt/wt) | Control | DAHP | *MA-GTPCH* | CP | *MA-GTPCH* plus CP | L-NAME | *MA-GTPCH* plus L-NAME |
| --- | --- | --- | --- | --- | --- | --- | --- |
| C16:0 | 14.81% | 17.60% | 13.67% | 19.26% | 13.48% | 23.14% | 15.71% |
| C18:0 | 11.16% | 9.60% | 12.52% | 8.06% | 13.27% | 9.33% | 14.21% |
| C18:1 | 19.56% | 30.21% | 11.64% | 41.08% | 30.49% | 29.53% | 26.58% |
| C18:2 | 6.95% | 6.50% | 7.28% | 5.95% | 6.91% | 7.63% | 7.00% |
| C18:3 | 4.72% | 4.91% | 3.75% | 3.94% | 3.90% | 5.94% | 5.40% |
| C20:3 | 4.18% | 3.00% | 3.50% | 1.65% | 4.71% | 2.76% | 3.50% |
| C20:4 | 32.63% | 22.36% | 42.43% | 16.71% | 23.26% | 18.13% | 24.95% |
